# Precise 3D modulation of electro-optical parameters during neurotransmitter uncaging experiments with neurons in vitro

**DOI:** 10.1038/s41598-020-70217-5

**Published:** 2020-08-07

**Authors:** Marco Cozzolino, Virginia Bazzurro, Elena Gatta, Paolo Bianchini, Elena Angeli, Mauro Robello, Alberto Diaspro

**Affiliations:** 1 DIFILAB, Department of Physics, University of Genoa, via Dodecaneso 33, 16143 Genoa, Italy; 2 Nanoscopy, CHT Erzelli, Istituto Italiano di Tecnologia, Genoa, Italy

**Keywords:** Biophysics, Neuroscience, Optics and photonics

## Abstract

Ruthenium–bipyridinetriphenylphosphine–GABA (RuBi–GABA) is a caged compound that allows studying the neuronal transmission in a specific region of a neuron. The inhibitory neurotransmitter γ-aminobutyric acid (GABA) is bound to a caged group that blocks the interaction of the neurotransmitter with its receptor site. Following linear—one-photon (1P)—and non-linear—multi-photon—absorption of light, the covalent bond of the caged molecule is broken, and GABA is released. Such a controlled release in time and space allows investigating the interaction with its receptor in four dimensions (X,Y,Z,t). Taking advantage of this strategy, we succeeded in addressing the modulation of GABA_A_ in rat cerebellar neurons by coupling the photoactivation process, by confocal or two-photon excitation microscopy, with the electrophysiological technique of the patch-clamp in the whole-cell configuration. Key parameters have been comprehensively investigated and correlated in a temporally and spatially confined way, namely: photoactivation laser power, time of exposure, and distance of the uncaging point from the cell of interest along the X, Y, Z spatial coordinates. The goal of studying specific biological events as a function of controlled physical parameters has been achieved.

## Introduction

The main inhibitory neurotransmitter of the mammalian central nervous system is γ-aminobutyric acid (GABA), which is present in almost 30% of synapses^1^.

GABA is responsible for the regulation of neuronal excitability: the modulation of the activity on its receptors is the mechanism of action of several molecules such as sedative, hypnotic, and antiepileptic drugs. GABA and other drugs like muscimol, THIP (gaboxadol) , and bicuculline have a binding site on GABA_A_ receptor, an ionotropic, pentameric receptor permeable to ion chloride. There is a large variability among GABA_A_ receptors due to the difference in subunit composition, causing different pharmacological properties. The subunit composition of GABA_A_ receptor subtypes is believed to determine their localization relative to the synapses and adapt their functional properties to the local temporal pattern of GABA impact, enabling phasic or tonic inhibition^2^. Specific and selective GABA_A_ drugs are essential tools for physiological and pharmacological localization among the receptor subtypes (populations)^3^. Recently, caged compounds were synthesized to control individual neuronal responses with a high spatial resolution^4^. The photorelease of caged bioactive molecules is a powerful tool for studying molecular mechanisms because such molecules can be delivered at precise and controllable instants of time.

These probe compounds are prepared via covalent appendage of a light-sensitive protecting group, called “cage”, to a signaling molecule that is unable to activate its target until the bond is broken by light^5^. Once uncaged, the molecule becomes active and it can interact with its receptor site inducing an outward current, due to the activation of GABA_A_ receptors^6^.

Uncaging by one-photon (1PE)^6–9^ or two-photon (2PE)^7,8,10–13^ excitation, coupled to patch-clamp, provide a useful technique to regulate the neurotransmitter release and to detect a selected biological target in a temporally and spatially confined way.

The use and relevance of caged compounds have been previously described in different papers^7,8,11,14–16^, but a comprehensive study is still lacking. It has already been reported that significant parameters are: laser power^7,9–11^, exposure time^12^, X–Y plane distance^6,8,11,13^, Z-axis distance^6,7^ from the cell body, and photoactivation method. These factors were analyzed in previous literature, for several GABA-linked compounds: 7-(dicarboxymethyl)-aminocoumarin–GABA^10^ (DCAC–GABA), (bis(2,2′-Bipyridine-*N*,*N*′)triphenylphosphine)-4-aminobutyric acid ruthenium hexafluorophosphate (RuBi–GABA)^6–9^, 7-[bis(carboxymethyl)amino]-4-methylcoumarin–GABA^7^ (BCMACM–GABA), 7-diethylamino coumarin–GABA excitable at 450 nm (DEAC450GABA)^8,11^ and 454 nm (DEAC454GABA)^12^.

This work intends to give an exhaustive view of the most significant parameters affecting uncaging experiments. We focus our attention on RuBi–GABA, a commercial compound which offers several advantages as reported by Rial Verde et al.^6^. We quantitatively investigate how all these factors affect the measurements and influence the modality and efficacy of GABA release and, consequently, the GABA_A_ response, a crucial point for understanding and interpreting delicate biological experiments. This analysis can provide useful information for decoupling the influence of experimental conditions from the observed neurophysiological events.

Moreover, in this work, we demonstrate how improving the localization precision, with advanced fluorescence-based optical methods, taking advantage of the intrinsic spatial localization provided by two-photon excitation fluorescence microscopy^17,18^.

In this way, caged GABA is topically released, in situ at a defined concentration and in a specific region of the neuronal cell, for mapping the localization and the functional distribution of GABA_A_ receptors. This allows studying the activation kinetics and pharmacology of GABA_A_ receptors in situ in cerebellar granule cells in vitro^10^.

## Materials and methods

### Animals

Sprague–Dawley rats were housed in the animal unit of the Department of Pharmacy, Section of Pharmacology and Toxicology of Genoa University. Experimental procedures and animal care complied with the EU Parliament and Council Directive of September 22nd, 2010 (2010/63/EU) and were approved by the Italian Ministry of Health (COD. 75F11.N.6DX) in accordance with D.M. 116/1992. All efforts were made to minimize animal suffering and to reduce the number of animals used for the experiments.

### Cell culture

Cerebellar granule cells were obtained from 6–8 days old Sprague–Dawley rats, as described previously^19^. They were plated at a density of 1.5–2.5 10^6^  cells/well coated with 20 μg/ml Poly-l-Lysine (Sigma-Aldrich, St. Louis, MO, USA).

The neurons were kept at 37 °C in a humidified 95% air/5% CO_2_ atmosphere and grown in 90% basal medium Eagle, 10% fetal calf serum (Sigma-Aldrich, St. Louis, MO, USA), 25 mM KCl, 2 mM glutamine and 100 µg/ml gentamicin.

At 18–24 h from plating, 10 μM cytosine arabinoside (Sigma-Aldrich, St. Louis, MO, USA) was added to the culture medium to prevent glial cell growth; at 48 h the medium was refreshed and 10 μM cytosine arabinoside was renewed. The cells were studied from the sixth to the tenth day in vitro.

### Electrophysiology

The experiments were performed in the whole-cell patch-clamp configuration with an Axopatch 200 B (Axon Instruments, Burlingame, CA, USA) as previously described by Robello et al.^20^.

The patch micropipettes (5–6 MΩ) were prepared using borosilicate glass capillaries (TW 150-3 World Precision Instruments Inc., Sarasota, FL, USA) and a P-30 puller (Sutter Instruments Co., Novato, CA, USA).

The currents were measured with a Labmaster D/A, A/D converter driven by pClamp 10 software (Axon Instruments, Burlingame, CA, USA) and analyzed with pClamp, SigmaPlot (SYSTAT Software, San Jose, CA, USA), MatLab (MathWorks, Natick, MA, USA).

The standard external solution (pH 7.4) used for the maintenance of the cells in the recording bath contained (in mM): 135 NaCl, 5.4 KCl, 1.8 CaCl_2_, 1 MgCl_2_, 5 HEPES, 10 glucose; the micropipettes were filled with the internal solution contained (in mM): 142 KCl, 10 HEPES, 2 EGTA, 2 MgCl_2_, 3 ATP and the pH was adjusted to 7.3 with Tris base.

The neurotransmitter GABA (Sigma-Aldrich, St. Louis, MO, USA) and the caged compound RuBi–GABA (Tocris Cookson Ltd, Bristol, UK) were diluted in an external solution at the needed concentration for experiments. Bicuculline was purchased from Sigma-Aldrich (St. Louis, MO, USA). Where not specified, the final concentration of RuBi–GABA used was 10 µM.

### One and two-photon imaging and uncaging

The uncaging procedure was performed by a three-channel Leica TCS SP5 laser-scanning confocal microscope equipped with 458-, 476-, 488-, 514-, 543-, and 633-nm excitation lines, through a plan-apochromatic oil immersion objective × 63/1.4. The images were acquired with the Leica “LAS AF” software package (Leica Microsystems, Germany).

A Ti:Sapphire laser (Chameleon II, Coherent Inc., Santa Clara, CA, USA) was used for two-photon excitation and uncaging, operating with pulses of 100 fs at the repetition frequency of 80 MHz. The laser power was measured at the objective focal point by a power meter (PM100A, Thorlabs Inc., Newton, NJ, USA). Laser stability was checked considering a balance between pulse width and power performances suggesting the utilization of 750 nm. Images in transmitted light were acquired using a 632.5 nm laser source coupled to a photomultiplier tube. For uncaging experiments, the FRAP Wizard Leica was used to set up the bleach points in terms of coordinates, power, and time. An image before and after the uncaging was taken to confirm the absence of relevant cell displacements.

## Results

Initially, to verify that uncaged RuBi–GABA caused the activation of GABA_A_ receptors, we carefully checked that the measured currents, after the photoexcitation of the caged compound, returned to the initial value.

**Figure 1:**
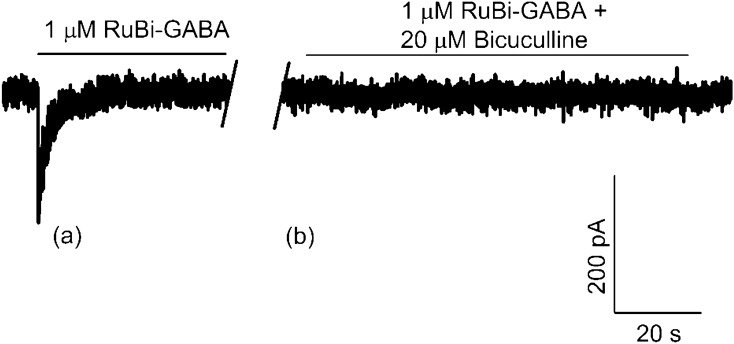
Current versus time. Current traces recorded, with a holding potential V = − 80 mV, on the same cell during the photoactivation of 1 µM RuBi–GABA by 1PE (458 nm, 100 ms, 6.7 µW) (**a**) and of 1 µM RuBi–GABA with 20 µM bicuculline uncaged by 1PE (same conditions) (**b**); no current spikes were registered.

Then, to make sure that photolyzed RuBi–GABA directly acted on GABA_A_ receptors, experiments with the competitive antagonist at the GABA-binding site bicuculline were performed (Fig. 1). GABA_A_ receptors were activated by 1 µM uncaged RuBi–GABA (left trace of Fig. 1), but, after the administration of 20 µM bicuculline, that occupies the GABA site in the channel, no significant current variations were recorded (right trace of Fig. 1). This further confirmed that, indeed, photoactivated RuBi–GABA precisely targets GABA_A_ receptors.

For characterizing the uncaging technique, we tested the main photophysical parameters that could influence the electrophysiological response: one and two-photon excitation (1PE and 2PE, respectively), laser power, exposure time, X-, Y-, Z-distance from the target cell and different holding potentials.

To get rid of variations due to cell-to-cell differences, we evaluated the effects of the variation of each photophysical parameter considering experiments performed on the same cell, and we treated the data, collected on different cells, with statistical methods.

### 1PE, 2PE and laser power

We investigated how the excitation method and laser power affect the electrophysiological response. It is well-known that one and two-photon uncaging methods produce excitation volumes with significantly different characteristics. In particular, when 1PE is used, the amount of compound, which is uncaged all along the laser optical path, is proportional to the light absorbed in each plane. A quantity that is overall conserved plane by plane following the distribution of the photon density.

On the other hand, 2PE uncaging is due to a simultaneous process of two-photon absorption that results in a volumetrically confined event. Therefore, the uncaging process is spatially localized.

For evaluating the effects of these intrinsically different mechanisms, we performed experiments spanning from low to high laser power both for 1P and 2P processes.

**Figure 2:**
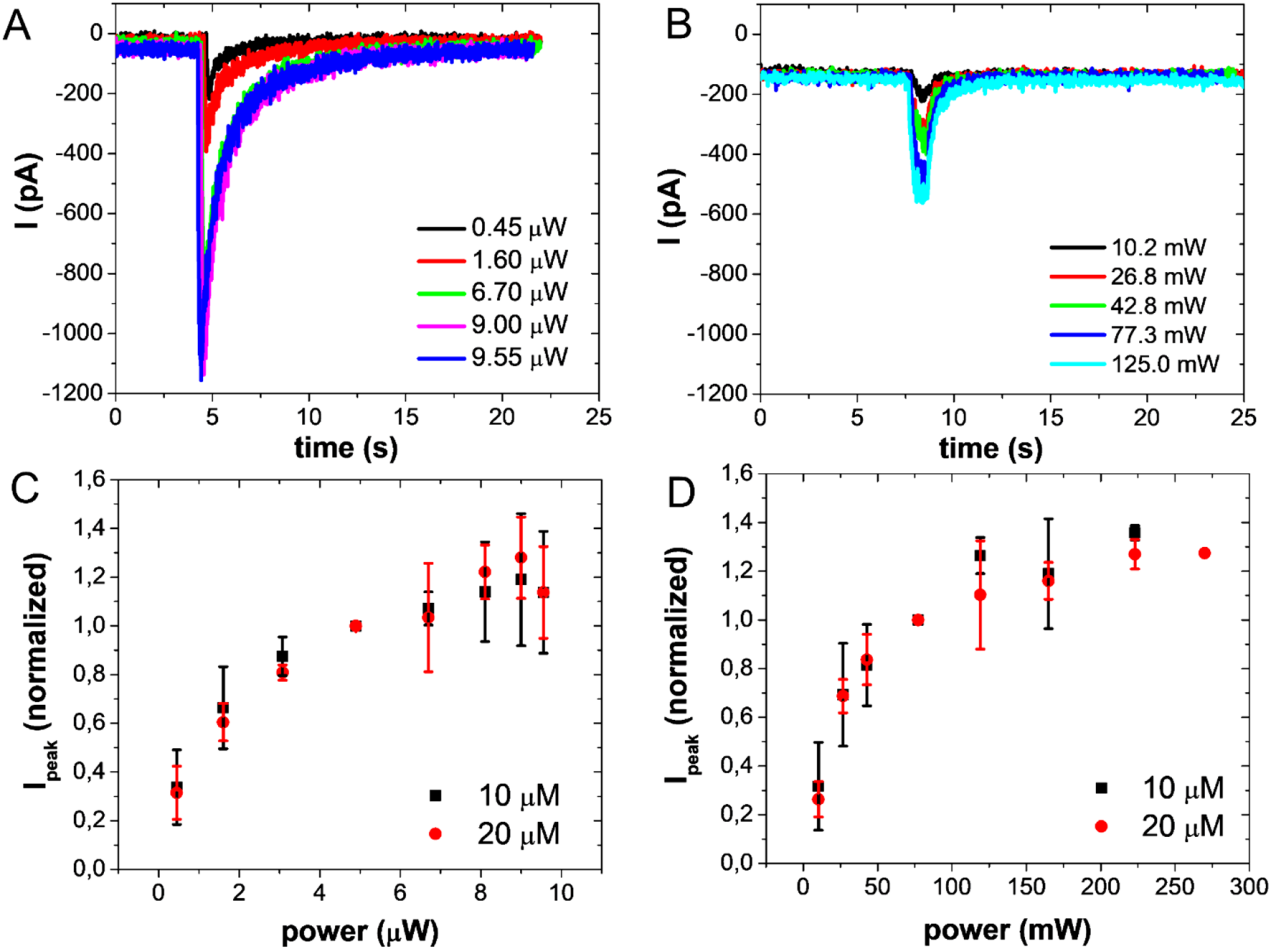
Current versus laser power. Examples of chloride current peaks evoked, on the same cell, by 10 µM RuBi–GABA uncaged at V = − 80 mV using different laser power for 1PE (458 nm, 100 ms) (**A**) and 2PE (750 nm, 100 ms) **(B**). Normalized values of current peaks evoked by 10 µM (black squares) and 20 µM (red dots) RuBi–GABA uncaged using different laser power for 1PE (458 nm, 100 ms) (**C**) and 2PE (750 nm, 100 ms) (**D**); the data are normalized to the peak current value evoked by using 4.9 µW and 77.3 mW for 1PE and 2PE, respectively. Each experimental value is estimated by calculating mean ± SEM on the data collected on at least three cells. For all the measurements the distance of the uncaging points from cell soma was below 1 micron.

In Fig. 2A,B, we report the currents measured after the activation of GABA_A_ receptors by one and two-photon uncaged RuBi–GABA, respectively. The current peaks due to 2P uncaging, result to be lower than the ones ascribed to 1P because of the localized volume.

Figure 2A,B show that saturation was reached following the power increase. This effect is related to the photoactivation of all the molecules in the excitation volume. Moreover, when raising the laser power (below the saturation regime), the amount of uncaged GABA increased, and this, in turn, implied an increase in the number of activated receptors. We also investigated the effects due to different compound concentrations. Figure 2C,D describe the normalized values of the peak current, as a function of the laser power, for 10 and 20 µM RuBi–GABA. The curves reached the plateau value for the same laser power. Hence, we deduced that peak current saturation was due to a limited number of uncaged molecules, and not to the saturation of the receptors. If this were the case, doubling the concentration of the available molecules (i.e., using 20 µm RuBi–GABA instead of 10 µM), saturation should occur at a lower laser power, but this was not the case since the curves had the same trend.

### Exposure time

We also studied the effects of the exposure time on the current profile both for 1PE and 2PE. The initial assumption was that uncaged molecules spread out of the excitation volume and diffuse in the solution. So, if the exposure time is longer than the time needed to diffuse out of the volume, uncaged molecules are replaced by fresh ones, diffusing back into the excitation volume. These molecules are, in turn, photoactivated; thus, for long exposure time, a kind of “cloud” of uncaged molecules is created around the uncaging volume.

**Figure 3:**
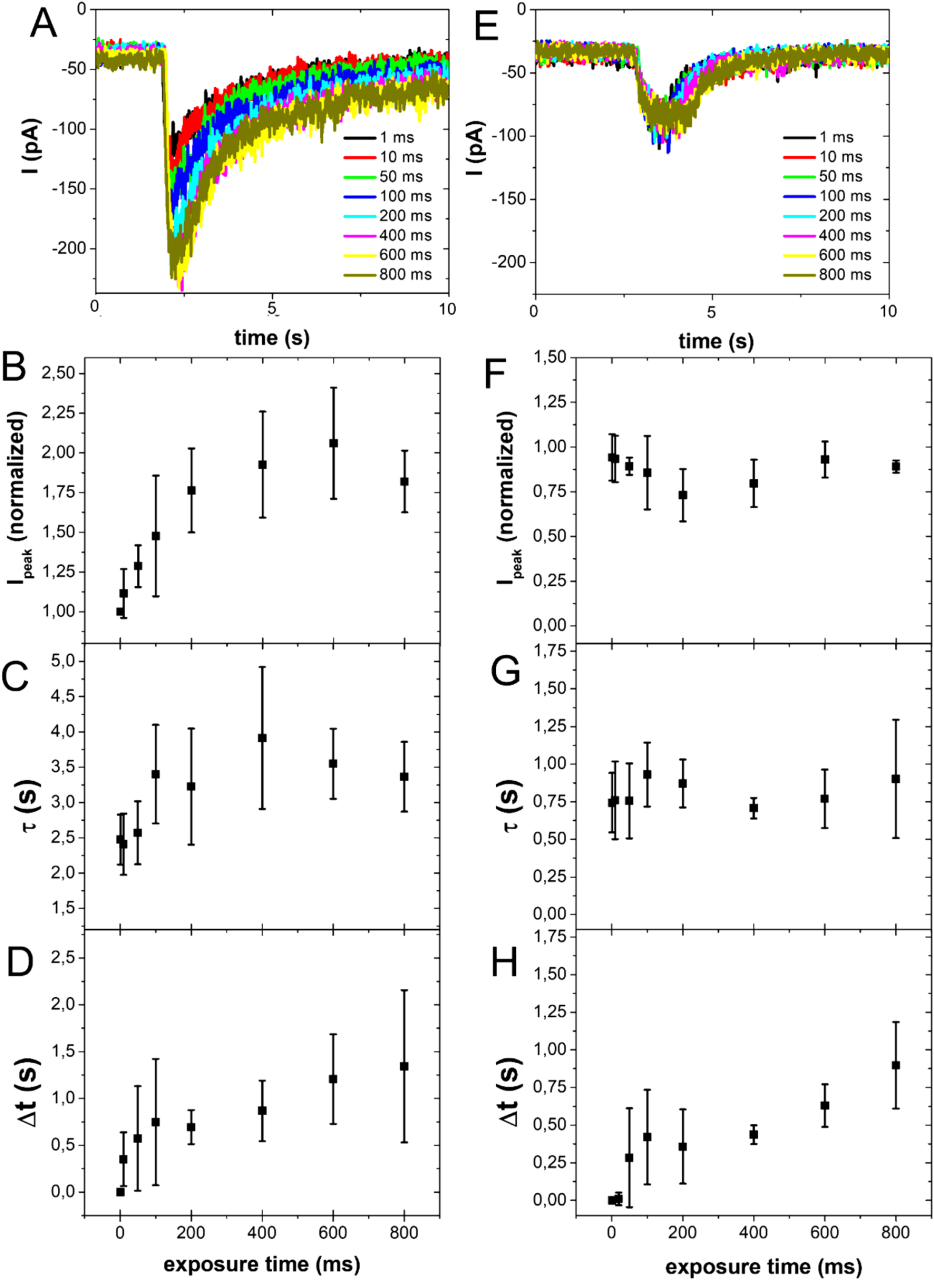
Current versus exposure time analysis. Examples of chloride current peaks evoked by 10 µM uncaged RuBi–GABA (V = − 80 mV) at different exposure times for 1PE (458 nm, 9.36 µW) (**A**) and 2PE (750 nm, 125 mW) (**E**) on the same cell. Normalized values of current peak evoked by 10 µM uncaged RuBi–GABA at different exposure times for 1PE (458 nm, 9.36 µW) (**B**) and 2PE (750 nm, 125 mW) (**F**), the values are normalized to the current measured for the lowest exposure time (1 ms). Time of decay as a function of the exposure time (τ) for 1PE (458 nm, 9.36 µW) (**C**) and 2PE (750 nm, 125 mW) (**G**). FWHM *versus* exposure time for 1PE (458 nm, 9.36 µW) (**D**) and 2PE (750 nm, 125 mW) (**H**). Each experimental value is estimated by calculating mean ± SEM on the data collected on at least 4 cells.

Experiments were performed (at least on 4 cells per session) progressively, changing the exposure time (1, 10, 50, 100, 200, 400, 600, 800 ms), for investigating how the electrophysiological measurements were affected (Fig. 3A,E).

Under a one-photon regime, increasing the exposure time, the intensity of the current peak (Fig. 3A,B) and the time of decay (Fig. 3C) raised to a plateau value. While considering the full width at half maximum (FWHM) of the signal, it resulted in a linear trend (Fig. 3D).

Under a two-photon regime, the behavior changed. In this case, the current peak and the time of decay did not vary (Fig. 3E–G), while the FWHM of the signal linearly rose when increasing the exposure time.

### X, Y, Z distance from the target

Analyzing how the X, Y, Z distance from the target affects the electrophysiological measurements is the very crucial point addressed in this study. Varying the distance of the uncaging point from the target, the amount of compound that binds GABA_A_ receptors on the plasma membrane may differ because of the isotropic diffusion of photolyzed molecules.

**Figure 4:**
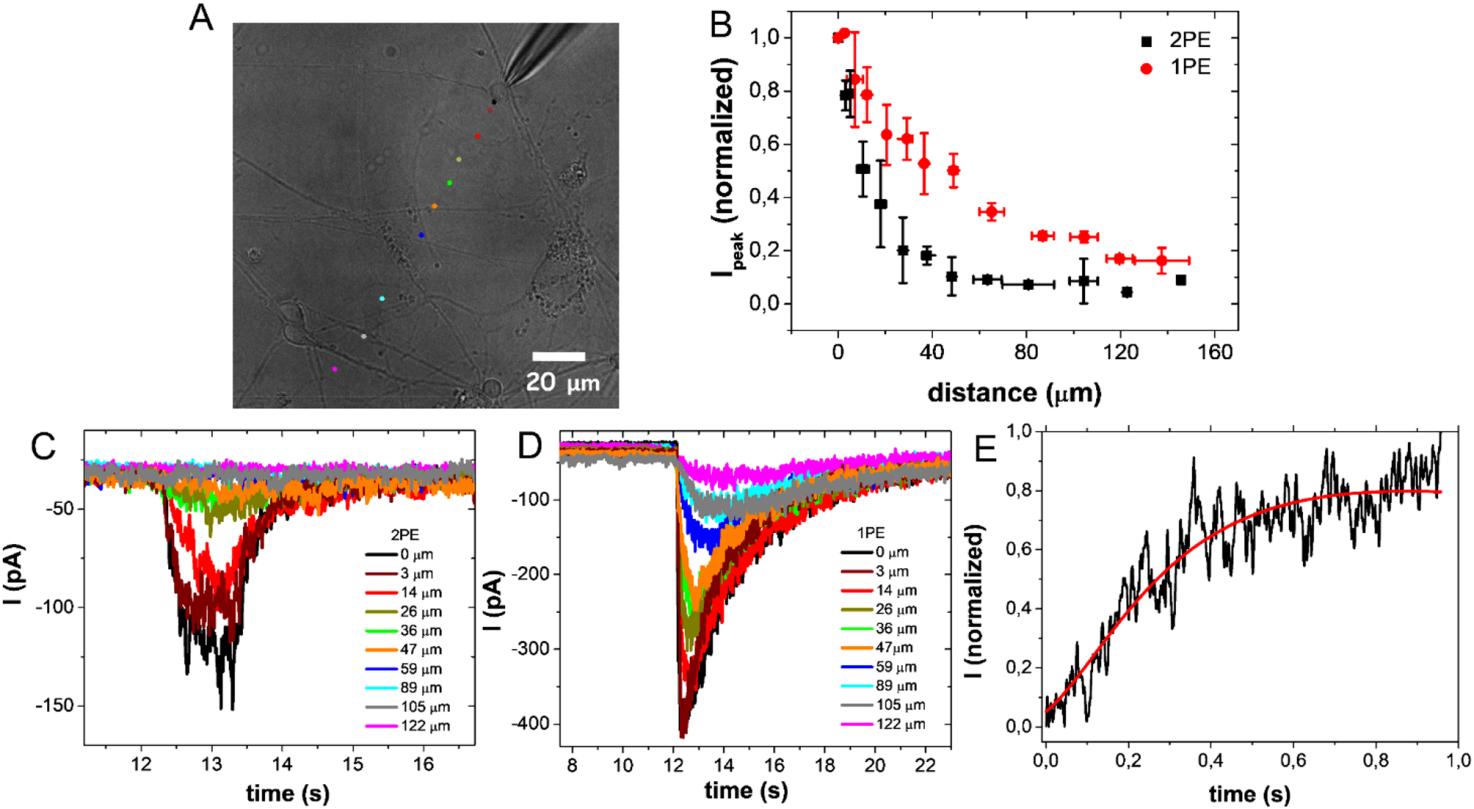
Current versus X–Y plane distance. Example of uncaging points with different distances from the target cell on the X–Y plane (**A**). Current peak values (average of data recorded for at least four cells) versus the distance from the target cell. Spikes were evoked by uncaging 10 µM RuBi–GABA at V = − 80 mV with 1PE (458 nm, 100 ms, 9.36 µW-red circles) and 2PE (750 nm, 100 ms, 77.3 mW-black squares). Current peak values are normalized to the current measured 0 µm far from the cell (**B**). Current peaks recorded for the uncaging points reported in (**A**) for 2PE (750 nm, 100 ms, 77.3 mW) (**C**) and 1PE (458 nm, 100 ms, 9.36 µW) (**D**). Example of rising current peak (black line) and fitting curve (red line) versus the time measured for an uncaging distance of 50 µm with 1PE (458 nm, 9.36 µW) (**E**).

Changing the uncaging distance on the X–Y plane (Fig. 4A), the measured current decreases during 1PE and 2PE (Fig. 4C,D).

In Fig. 4E, it is shown the rising current peak, as a function of time, during 1PE at 50 μm uncaging distance; when the distance from the target increases, the rise time becomes longer (Fig. 4C,D).

The molecule diffusion dynamics can be approximated as the diffusion of an instantaneous point source:

1 $$ C\left( {x,t} \right) = \frac{A}{{\sqrt {4\pi D \cdot t} }}e^{{\left( { - \frac{{x^{2} }}{4Dt}} \right)}} , $$

where C is the molecule concentration, D the diffusion coefficient, x the distance from the point source, t the time and A an arbitrary constant^21^.

The current rise time was fitted with Eq. 1.

The calculated diffusion coefficient was (6 ± 3) 10^−10^ m^2^ s^−1^, a value in agreement with the theoretical value estimated for GABA, considering a temperature of 25 °C and the viscosity of water of 1 mPa s. The result was confirmed by the analysis of the data acquired for four distance values and four different cells.

**Figure 5:**
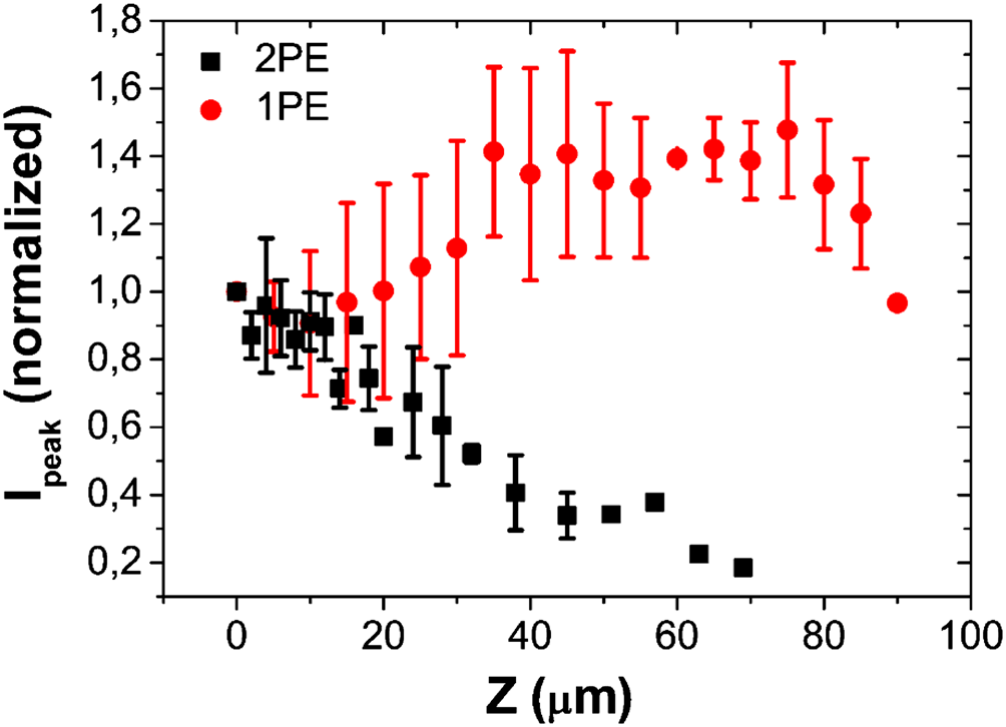
Current peak versus Z-axis distance. Current peak values (average of data recorded for at least three cells) versus distance on Z-axis evoked by uncaging 10 µM RuBi–GABA at V = − 80 mV for 1PE (458 nm, 100 ms, 9.36 µW-red circles) and 2PE (750 nm, 100 ms, 77.3 mW-black squares). Current peaks are normalized to the value recorded close to the surface of the cover slip (Z = 0 µm).

Figure 4B shows the current peak values, as a function of the distance from the cellular membrane, during the experiments performed with 1PE (red dots) and 2PE (black squares). When uncaging occurs in a confined volume (i.e. with 2PE), the current peaks show a faster decrease compared to 1PE, as the distance on the X–Y plane increases. Whereas, for uncaging at different positions on Z-axis (Fig. 5), current peaks measured for 2PE and 1PE have different trends.

With 2PE, the current peak value remains constant within a distance of 10 µm from the coverslip surface (a distance comparable to the size of a cerebellar granule cell), farther the current peak values linearly decrease.

The situation is different for 1PE: in fact, the current does not show a diminishing trend, but it remains constant (red dots of Fig. 5).

### Current–voltage curves

**Figure 6:**
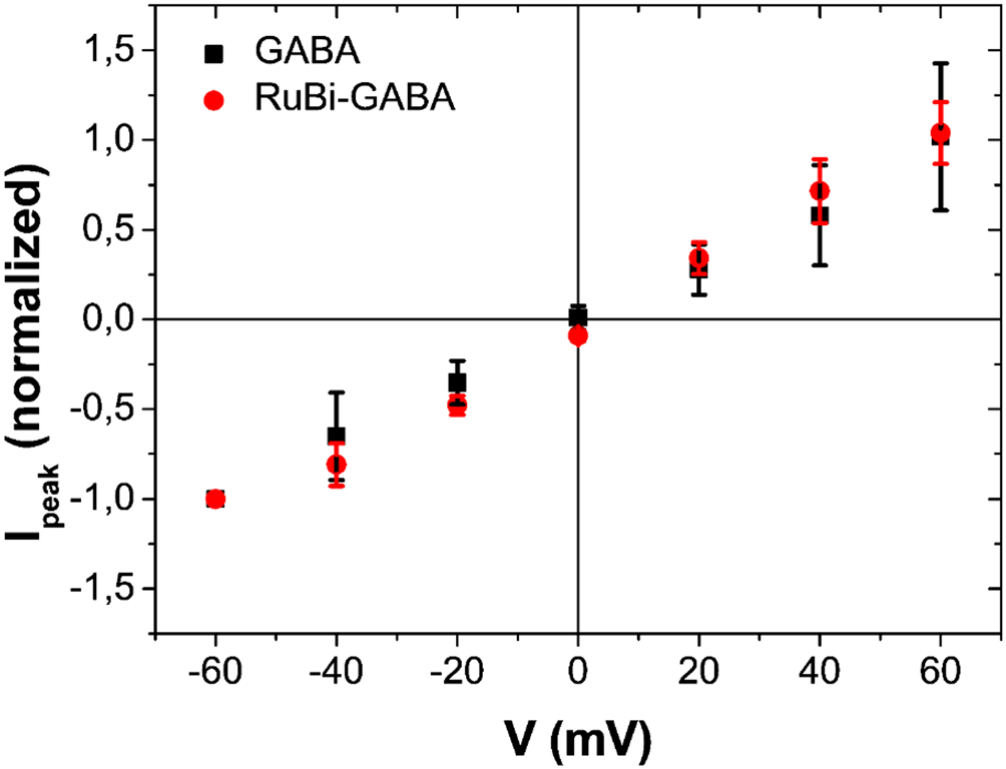
Current–voltage (IV) curves. IV curve measured after the administration of 10 µM GABA (black squares) and after uncaging 10 µM RuBi–GABA with 1PE (458 nm, 100 ms, 9 µW-red circles). Current peak values (average of data recorded for at least four cells) are normalized to the current peak recorded for − 60 mV.

We have performed experiments at different holding potentials for investigating if and how neurotransmitter GABA and uncaged RuBi–GABA had different effects on current peak values; the current–voltage (I–V) curve measured with 10 µM GABA shows an ohmic trend (Fig. 6) as previously described by Robello et al.^22^.

Other experiments were carried out with photolyzed 10 µM RuBi–GABA (1PE, 458 nm, 9 µW, 100 ms). The I–V curve was recorded applying a voltage bias spanning from − 60 mV to + 60 mV with steps of 20 mV. This curve, reported in Fig. 6, shows an ohmic behavior similarly to neurotransmitter GABA.

## Discussion

In recent decades, the uncaging method has been developed to study electrophysiological processes in a well-defined time and position near the cell^10^. In this work, we show how the photoactivation of caged RuBi–GABA allows the release of a definite amount of uncaged GABA in a specific location in the proximity of a target cerebellar granule cells. Furthermore, we investigate how physical parameters could affect the neuronal response when GABA is administered by uncaging. When electrophysiological measurements are coupled with the uncaging method, to study, for example, the neuronal response to drugs, it is important to distinguish and understand how changes in photophysical conditions, such as the laser uncaging intensity, distance from cell soma, etc., could affect the biological response.

We initially verified that the measured current was actually due to the activation of GABA_A_ receptors, as demonstrated by experiments carried out using bicuculline (Fig. 1), which occupies the GABA site in the channel avoiding the binding of the neurotransmitter with its receptor. Then, we compared and analyzed current curves measured by changing different physical parameters, both in terms of peak values and signal shape, in order to identify the best conditions to set during the experiments.

The differences in the current peaks, shown in Fig. 2A,B, arise from the different number of photolyzed molecules which is larger under 1P regime than 2P, as 1P results in a double cone interaction volume around the focal point, while 2P regime confines the uncaging within a localized ellipsoid^23^. Changes in the laser intensity may cause a variation in the number of uncaged molecules either for 1P and for 2P processes (Fig. 2C,D). In fact, for low beam power, few molecules are released as expected. If we gradually increase the laser power, the number of uncaged GABA increases until all the molecules in the excitation volume are photolyzed. Therefore, only the number of released molecules changes, but not the uncaging precision.

It is possible to change the administering time of uncaged molecules modifying the laser exposure time. The variation of this parameter causes different effects when used in one-photon or two-photon regimes, as shown by the different trends of the graphs reported in Fig. 3.

The increase of the exposure time for 1P results in a larger uncaging volume and produces an increase in the current intensity (Fig. 3B). This is due to the fact that the photon density in those optical planes, far from the focal point, is small and the probability of uncaging molecules, interacting with the receptors, is low. For longer exposure time, the probability rises, causing the increase of the excitation volume and of the number of uncaged molecules. Thus, a larger number of receptors is involved in the current signal generation and, consequently, the decay time rises (Fig. 3C).

Also, the full width at half maximum (FWHM) of the current trace increases due to an interplay of two effects: a longer decay time and the administration of the neurotransmitter for a longer extent of time (Fig. 3D). Under a 2P regime, the behavior changes as expected. In fact, as shown in Fig. 3F,G, the current intensity and the decay time remain constant, as the 2P excitation volume does not increase with the excitation time, therefore the amount of receptors involved in the generation of the signal is constant.

The FWHM of the current peaks linearly increases with the exposure time, as the neurotransmitter is administered for a longer time, as shown in Fig. 3H. Taking into account the distance changes along X, Y and Z-axis, two-photon excitation is more localized than one-photon.

2P uncaging, as demonstrated for answering other biophysical challenging questions^24^, is crucial for definend the volume where photointeractions take place to connect them with related biological events subject to molecular trafficking.

Varying the uncaging point on X–Y plane (Fig. 4A) and moving away from the target, the amount of molecules, reaching the receptor gradually decreases, due to the isotropic diffusion of the compound in solution. 2P uncaging effect results more sensitive to the distance variations compared to 1P (Fig. 4B).

The increase of the uncaging distance fits well with a longer signal rise time due to the fact that molecules, uncaged farther from the cell, need more time to reach the target (Fig. 4C–E).

The theoretical value of GABA diffusion coefficient, evaluated for GABA molecules with the Stokes–Einstein equation, considering water as medium and a concentration of 10 µM, is 7 10^−10^ m^2^ s^−1^.

The value obtained analyzing the experimental data, as shown in Fig. 4E, [(6 ± 3) 10^–10^ m^2^ s^−1^], results to be consistent with the theoretical value. Therefore, the variation of the peak rise current is due to diffusion phenomena.

Along the Z-axis the 2P-uncaging effect is reported (Fig. 5, black squares). The value of the current peak remains approximately constant within the first 10 μm then it diminishes as distance increases. The reason is that cerebellar granule cells have a size of nearly 10 μm, so the current remains constant when the uncaging point is close to the receptors on the plasma membrane. While, when the uncaging point is moved far from the cell in the Z-axis direction, the number of photolyzed molecules reaching the receptors, decreases because of diffusion, similarly to changes of uncaging distance in the X–Y plane.

During 1P experiments (see Fig. 5, red circles), when moving the uncaging point, from 0 to 40 µm away from the cell, an increase of the current peaks is detected, this is due to the displacement, within the solution, of the low excitation cone that, initially (for Z = 0 µm), is under the glass slice. In fact, as this cone interacts with larger solution volume, the number of uncaged molecules increases. For longer uncaging distances, i.e. from 40 to 80 µm, the effects due to variations in the interaction volume of the lower and upper illumination cones with the solution balance and the current peaks reach a plateau value.

**Figure 7:**
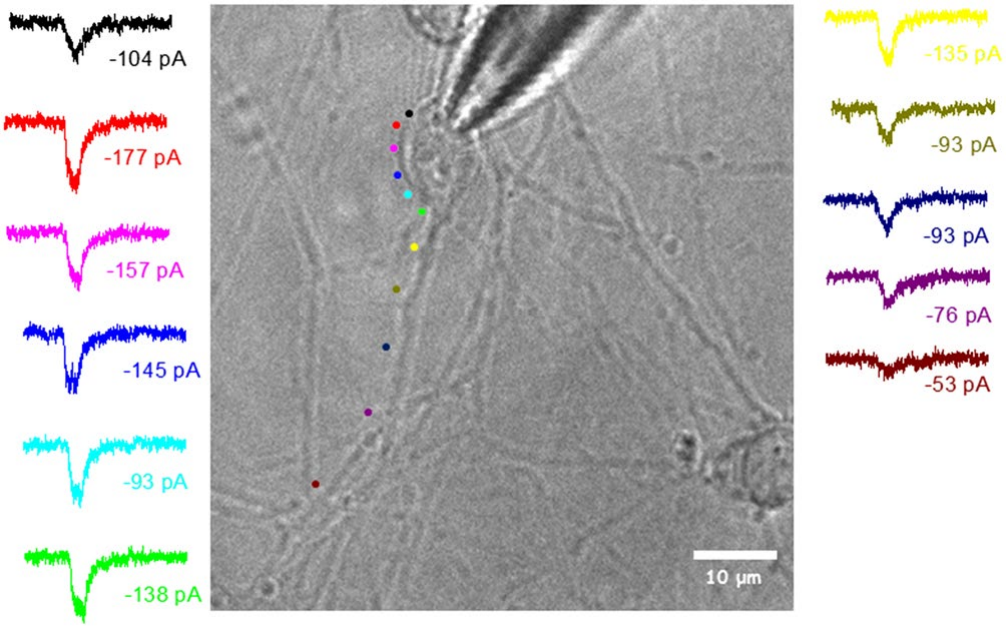
Map of uncaging points. Map of the current traces (with the corresponding peak value) recorded uncaging 10 µM RuBi–GABA (V = − 80 mV) at different points of the neuron for 2PE (750 nm, 100 ms, 77.3 mW). The color of the current trace and the dot, indicating the uncaging point on the optical image, is the same.

The relevance of this technique for investigating the neurophysiological response of different regions of the neuron is demonstrated by the map reported in Fig. 7. While changing the position of the photoactivation point along the neuron, we registered the electrophysiological currents evoked by uncaging a solution of 10 µM RuBi–GABA. The significant decrease of the current peak values, recorded while moving away from the cell body, suggests that the receptors’ density in the neuronal extensions is lower than in the soma.

## Conclusion

The use of caged compounds with patch clamp technique and coupled to the possibility of uncaging molecules by localized photo-processes is a powerful biophysical approach for investigating the distribution of receptors on neuronal cells.

Such a methodological combination allows electrophysiologically examinating a confined area of the cells, enabling researchers to define detailed maps on kinetics, physiology, and pharmacology of specific molecules. In our case, we focused the attention to pentameric GABA_A_ receptor of different regions of the neuron, such as the cell body, axons or dendrites to contribute to the development of drugs that specifically affect the GABAergic system, for example sites of action of benzodiazepines,barbiturates, and neurosteroids.

Comparison of 1P and 2P based uncaging processes allows refining protocols, an essential trace, for those experiments related to the understanding of specific mechanisms related to neuronal cell communications. The three-dimensional localization ability intrinsically provided by 2P imaging and excitation also has the added value of defining threshold doses of pharmacological treatments.

So, to take full advantage of the possibilities offered by this technique, it is necessary to know, in detail, how the measurement conditions affect the neurophysiological response. With this goal in mind, we analyzed how the physical parameters used during the uncaging process (exposure time, laser power, uncaging point, etc.) influence the electrophysiological signals recorded during the activation of GABA_A_ receptors. Moreover, we compared and analyzed experimental data recorded photoactivating a solution of RuBi–GABA, a caged compound, under 1P and 2P regimes.

Finally, we used this measurement method to map the electrophysiological response of GABA_A_ receptors in different regions of the neuron, inferring that receptor density in the soma is higher than in the extensions.

The technique, we have extensively investigated here, is a promising tool for a variety of applications, including the pharmacological development of new drugs; in fact, it can be used to study how the receptor density may change in a precise area after a pharmacological treatment such as benzodiazepines administration.These drugs are one of the most widely prescribed pharmacologic agents are used for numerous indications, including anxiety, insomnia, panic disorders, alcohol withdrawal, muscle relaxation, and epilepsy. Quantitative control of dose parameters plays a fundamental role when designing a pharmnacological treatment since there are significant physical, mental and social risks associated with the long-term use of benzodiazepines.

The use of 2P uncaging and the control of the different parameters involved is also related to the challenging perspective of translating such an approach to studies based on the use of three-dimensional organoids^25^.

## Supplementary information

Supplementary file1
