# Supplementary material for: Precise 3D modulation of electro-optical parameters during neurotransmitter uncaging experiments with neurons in vitro
Source: Sci Rep. 2020 Aug 7;10:13380. doi: 10.1038/s41598-020-70217-5 (PMC7414112; doi:10.1038/s41598-020-70217-5)
Supplement: Supplementary file 1 — Supplementary file1 [file 41598_2020_70217_MOESM1_ESM.docx]

Supplementary Information

Precise 3D modulation of electro-optical parameters during neurotransmitter uncaging experiments with neurons *in vitro*

*Marco Cozzolino^1,2,§^, Virginia Bazzurro^1,§^, Elena Gatta^1^, Paolo Bianchini^2^, Elena Angeli^1^, Mauro Robello^1^, Alberto Diaspro^1,2,*^*

(1) DIFILAB, Department of Physics, University of Genoa, via Dodecaneso 33, 16143 Genoa, Italy

(2) Nanoscopy, CHT Erzelli, Istituto Italiano di Tecnologia, Genoa, Italy

KEYWORDS**:** confocal microscopy, two-photon excitation microscopy, 3D localized uncaging, patch-clamp, RuBi-GABA, GABA_A_ receptors, cerebellar granule cells, linear and nonlinear uncaging.

*§* Equally contributing authors

*corresponding author: diaspro@fisica.unige.it


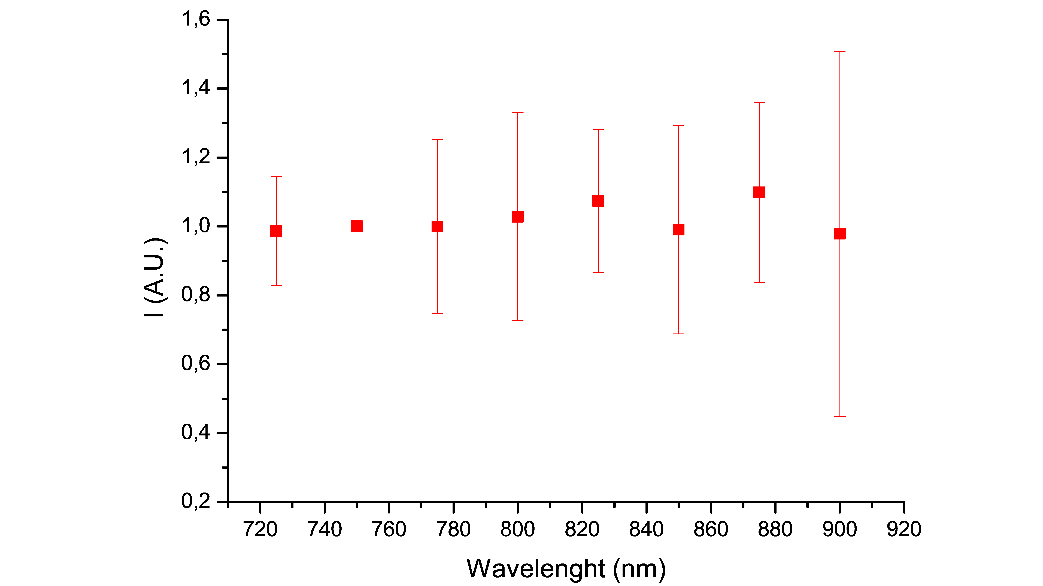


Fig. S1: Current peaks measured by uncaging 10 µM RuBi-GABA at different wavelengths using 2PE (100 ms, 45 mW, voltage clamp at -80 mV) on cerebellar granule cells.

We performed experiments by using different wavelengths (see Fig. S1), and the normalized peak currents do not show significant differences in the range investigated.

Thus, we chose to uncage RuBi-GABA at 750 nm as it represents a good value in terms of spatial resolution, i.e. uncaging volume. It is an acceptable volume also in terms of spatial localization^17,23^ when electrophysiologically mapping the cellular response.


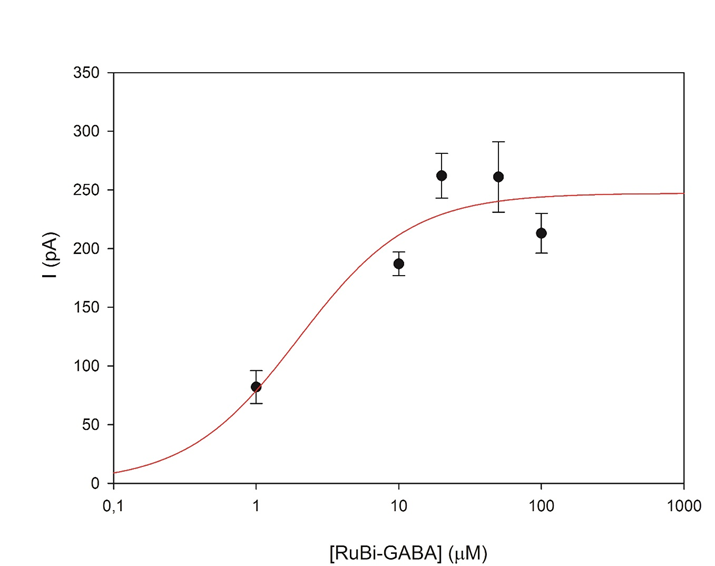


Fig. S2 Dose-response curve of RuBi-GABA measured by using 2PE (λ=750 nm, 100 ms, 45 mW, voltage clamp at -80 mV) on cerebellar granule cells. Semilogarithmic plot of peak amplitude as a function of RuBi-GABA concentration. Theoretical fitting for peak values is obtained from Eq. 1.

Fig. S3 Concentration of excited molecules *versus* laser power. Curve traced comparing the experimental values of the current peaks with the dose-response curve Fig S2.
